# Decreasing pH Results in a Reduction of Anthocyanin Coprecipitation during Cold Stabilization of Purple Grape Juice

**DOI:** 10.3390/molecules20010556

**Published:** 2015-01-05

**Authors:** David C. Manns, Passaporn Siricururatana, Olga I. Padilla-Zakour, Gavin L. Sacks

**Affiliations:** 1 Department of Food Science, New York State Agricultural Experiment Station, Cornell University, 630 W. North St., Geneva, NY 14456, USA; 2 Department of Food Science, Cornell University, Stocking Hall, Ithaca, NY 14853, USA

**Keywords:** cold stabilization, anthocyanin, potassium bitartrate, coprecipitation

## Abstract

Anthocyanin pigments in grape juice can coprecipitate with potassium bitartrate (KHT) crystals during cold stabilization, but factors that reduce these adsorptive losses are not well understood. We hypothesized that coprecipitation on a % w/w basis should be decreased at lower pH. In initial experiments, model juice solutions containing an anthocyanin monoglucoside extract and varying pH values were subjected to cold-storage to induce KHT crystallization, and anthocyanins in the resulting precipitant were characterized by HPLC. The pH of the model juice was directly correlated with the % w/w concentration of anthocyanins in the KHT crystals, with a maximum observed at pH 3.40 (0.20% w/w) and a minimum at pH 2.35 (0.01% w/w). A pH dependency was also observed for anthocyanin-KHT coprecipitation in purple Concord grape juice, although the effect was smaller. Coprecipitation was significantly greater for anthocyanin monoglucosides and acylated anthocyanins as compared to anthocyanin diglucosides at pH > 3.05, but coprecipitation of mono- and acylated forms declined more sharply at lower pH values.

## 1. Introduction

While a wide range of phenolic compounds have been detected in juices from Concord grape juices and related grape cultivars, including hydroxycinnamates, flavan-3-ols, flavonols, and stilbenes (e.g., resveratrol) [1,2], the major species are the anthocyanins [1]. These anthocyanins are the major compounds responsible for the pigmentation of red and purple grapes and are critical to consumer acceptance of grape-derived products like juices and wines [3]. Additionally, the anthocyanins along with other polyphenols and their metabolites have been implicated as important phytonutrients capable of reducing the incidence of chronic disease [4,5]. Due to their overall importance to the acceptability of fruit juices and related products, several publications have considered the impact of production practices on anthocyanin stability, particularly acid-catalyzed hydrolysis and polymerization reactions [6,7,8].

In grape juice and wines, an additional source of anthocyanin losses during production is coprecipitation of anthocyanins with potassium bitartrate [9,10,11,12]. Grapes are uniquely high in tartaric acid compared to other fruits, 2–14 g/kg, or 0.01–0.07 M [13], and also contain high concentrations of potassium, 0.01–0.06 M [14,15]. These concentrations are at or above the solubility of potassium bitartrate (KHT) in pure water at 0 °C (0.01 M), although the apparent solubility of KHT in real juices is higher due to the presence of polyphenols, polysaccharides, and other constituents that can inhibit crystallization [16,17]. To prevent formation of KHT crystals in finished products, a cold-stabilization step is usually performed on grape juices and wines prior to bottling [18].

The factors affecting the kinetics and thermodynamics of KHT precipitation are well studied [19,20,21]. Coprecipitation of anthocyanins with KHT resulted in a 20%–40% loss of total anthocyanins from Concord juice in one report [9], with similar losses reported elsewhere [10,12]. The loss of anthocyanins during cold stabilization is comparable to gains achieved by widely used juice processing treatments such as the use of pectolytic enzymes [22] or thermal treatments [23]. Thus, elimination of anthocyanin coprecipitation could be considered an unexploited route to increasing final anthocyanin concentrations in grape juice. The coprecipitation process also results in enrichment of the anthocyanins in the KHT precipitate compared to the remaining solution by about an order of magnitude [10,12]. Coprecipitation of tannins, hydroxycinnamic acids, flavonols, and other organic compounds are also reported to occur, with preferential loss of less polar species [12].

The mechanism for the loss of anthocyanins during cold stabilization is not well understood. Occlusion of anthocyanins within the crystal is unlikely to occur, as the proportions of coprecipitating compounds are different than their proportions in solution [16]. Rather, the interaction of anthocyanins and KHT appears to be adsorptive in nature [11], a process which also inhibits crystal growth, changes crystal morphology, and increases the apparent solubility of KHT in grape products *vs.* pure water [16]. The interactions between the KHT crystal face and phenolics are variously proposed to be ionic, hydrogen-bonding, or charge-transfer in nature [24,25]. X-ray crystallography data indicates that the {010} face is populated by the bitartrate species, and it was hypothesized that this would result in a positive surface charge on this face created by excess potassium ions, and consequentially the adsorption of Lewis bases, e.g. the neutral forms of anthocyanins [25]. In contradiction, Alongi *et al.* [10] observed that anthocyanin species which favored the flavylium cation form (lower pK_h_ value) were more likely to be lost via coprecipitation, indicating that the anthocyanins may interact directly with bitartrate at the surface.

Regardless of the mechanism, the loss of anthocyanins and other polyphenolics during KHT precipitation is undesirable to the wine and grape industries, but strategies to reduce these losses are largely unknown. A study by our group showed that anthocyanin coprecipitation is significantly less in juice concentrate (59 Brix) as compared to single strength juice [10]. Cold-stabilization of single-strength Concord juice prior to concentration resulted in moderate losses (~20%) of anthocyanins, similar to previous reports, while concentration prior to cold-stabilization (so-called “direct to concentrate”) resulted in no significant loss of anthocyanins. Compositional analysis of KHT crystals yielded similar results—although comparable losses of KHT occurred in both systems, the precipitate from the direct to concentrate had lower anthocyanin content (0.13% *vs.* 0.80% w/w). The improved anthocyanin stability achieved in concentrate did not appear to result from increased co-pigmentation. Because anthocyanin species that existed more in charged forms (higher pK_h_ values) were more likely to coprecipitate, it was hypothesized that the reduction in coprecipitation in concentrate could be credited to the lower pH of concentrate. The pH of concentrate (2.5) is lower than single-strength juice (pH = 3.1), which should result in a neutralization of the surface charge of the KHT surface [24]. However, because concentrate differs from juice in many other respects (greater ionic strength, lower water activity, *etc.*), this was not conclusive.

In this work, we investigated if a pH decrease could induce changes in the degree of coprecipiation of anthocyanins with potassium bitartrate during grape juice cold-stabilization. The current study investigated the effects of pH on coprecipitation of anthocyanins with KHT, in both a model juice system and a purple Concord grape juice.

## 2. Results and Discussion

### 2.1. Anthocyanin-Bitartrate Coprecipitation in a Model Juice

**Figure 1:**
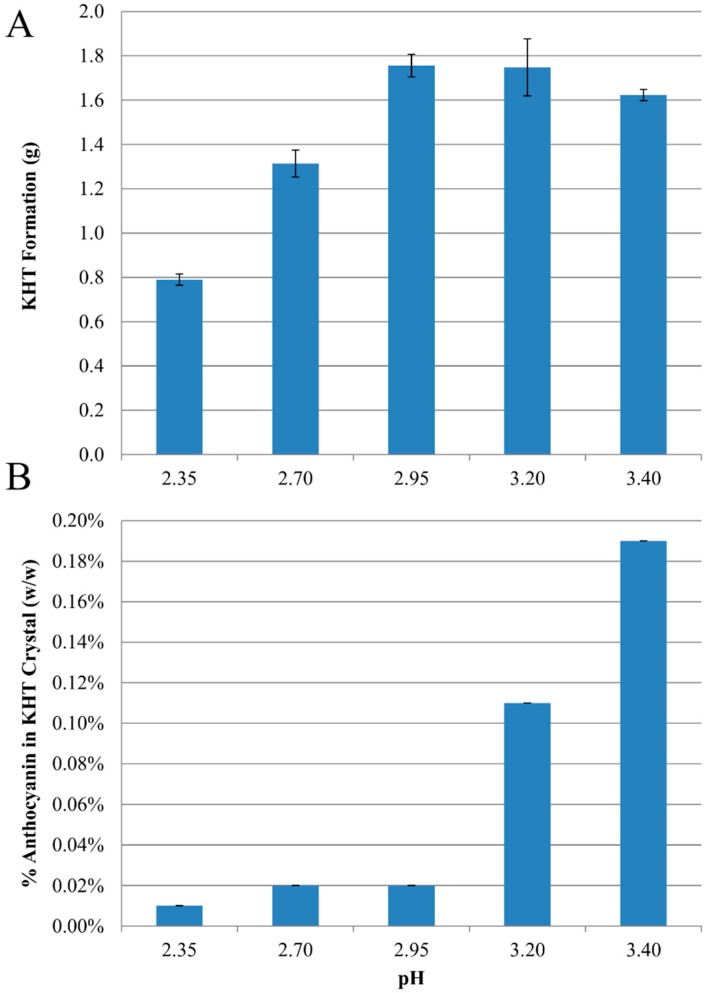
Effects of pH on (**A**) mass (g) of KHT crystals recovered by filtration after cold stabilization of blackcurrant model juices, and (**B**) total anthocyanin (% w/w) coprecipitating with KHT crystals recovered from cold stabilized blackcurrant model juice.

As an initial evaluation of this hypothesis, a model juice containing a blackcurrant anthocyanin extract was prepared with potassium (0.02–0.04 M) and tartaric acid (0.02–0.04 M) concentrations within the range ordinarily encountered in grape juice: 0.01–0.06 M for potassium [14,15] and 0.01–0.07 M for tartaric acid [13]. Initial concentrations of the four primary anthocyanins present in the model blackcurrant juice were 46.35, 161.1, 32.38, and 197.2 µg/mL malvidin-3-glucoside equivalents for delphinidin-3-glucoside (D-3-G), delphinidin-3-rutinoside (D-3-R), cyanidin-3-glucoside (C-3-G), and cyanidin-3-rutinoside (C-3-R), respectively. The pH range, 2.35–3.40, was selected to bracket the range typically observed in single strength grape juice (pH = 3.0–3.5) as well as in 59 Brix juice concentrate (pH = 2.5). We observed negligible KHT precipitation in most treatments with 0.02 M tartaric acid and/or potassium (data not shown), and characterization of anthocyanin content in the resulting crystals was not feasible. As a result, only data for 0.04 M K^+^ and 0.04 M tartaric acid with varying pH are reported. During cold storage of model juices we observed precipitation of KHT (Figure 1A), with pH 2.95–3.40 model juices precipitated 1.62–1.76 g (8.63–9.33 mmoles) of KHT, significantly greater than the mass precipitated at pH 2.35 (0.790, or 4.2 mmoles) and pH 2.70 (1.31, or 6.98 mmoles). Anthocyanins in KHT crystals were quantified by HPLC following redissolution, and the concentration of anthocyanin in KHT crystals (%, w/w) as a function of pH is shown in Figure 1B. The highest anthocyanin concentration was 0.19% w/w at pH 3.4, and decreased to a low of 0.01% w/w at pH 2.35.

**Figure 2:**
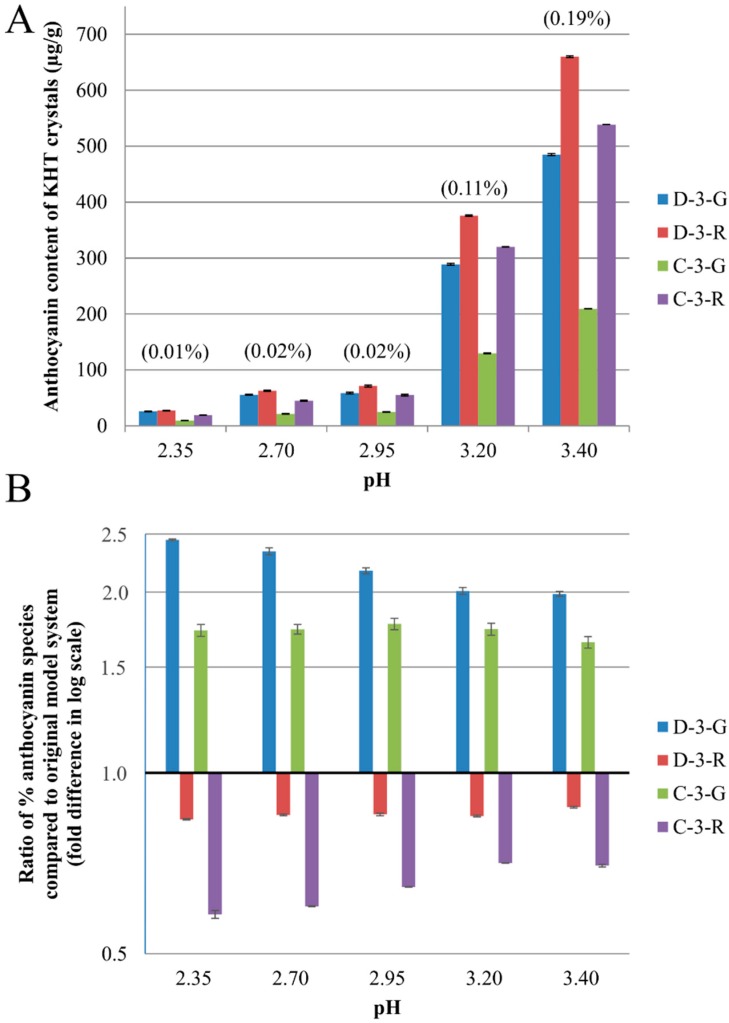
(**A**) Anthocyanin concentration (μg/g) in KHT crystals as a function of pH recovered from model juices containing 0.04 M tartaric acid and 0.04 M KCl. The number in parenthesis represented the total anthocyanin in KHT crystals (% w/w); (**B**) Selectivity of coprecipitation, calculated as the ratio of an anthocyanin species concentration in KHT crystals (normalized to total anthocyanin in KHT crystal) to the anthocyanin species concentration in original model juice (normalized to total anthocyanin in original juice). D-3-G: delphinidin-3-glucoside; D-3-R: delphinidin-3-rutinoside; C-3-G: cyanidin-3-glucoside; C-3-R: cyanidin-3-rutinoside.

Decreasing pH resulted in decreased coprecipitation for all four black currant anthocyanins (Figure 2A), and significant differences (*p* < 0.05 by ANOVA) in the relative losses of each species were also observed (Figure 2B). The delphinidin- and cyanidin-3-glucoside were enriched by a factor of 1.5 to 3, while the corresponding rutinosides were depleted by 10%–50%.

### 2.2. Anthocyanin-Bitartrate Coprecipitation in a Purple Concord Grape Juice

The model juice study was duplicated with a real Concord juice adjusted to one of six pH values prior to cold-stabilization. The anthocyanin composition, organic acid composition, and basic juice chemistry of the original juice are reported in Table 1.

**Table 1:** Initial anthocyanin concentrations and basic juice parameters in Concord juice.

Analyte	Concentration ^a^
*Anthocyanin Diglucosides*	
Del-3,5-Di	26.0 (0.38)
Cy-3,5-Di	32.0 (0.63)
Pet-3,5-Di	16.4 (0.65)
Peo-3,5-Di	33.9 (0.68)
Mvn-3,5-Di	30.2 (0.72)
*Anthocyanin Monoglucosides*	
Del-3-Glu	94.8 (1.0)
Cy-3-Glu	74.4 (0.82)
Pet-3-Glu	28.4 (0.65)
Peo-3-Glu	13.7 (0.37)
Mvn-3-Glu	19.2 (0.59)
*Acylated Anthocyanins*	122.6 (1.3)
**Total Anthocyanins**	491.6 (0.84)
*Organic Acids*	
Citric Acid	0.23 (0.01)
Tartaric Acid	11.5 (0.07)
Malic Acid	2.38 (0.03)
Total Soluble Solids	20.0 (0.1)
Initial pH	3.05

^a^: All anthocyanin units are in mg/L. The diglucosides are expressed in malvidin-3,5-diglucoside equivalents. The monoglucosides and modified anthocyanins are expressed in malvidin-3-glucoside equivalents. Organic acids are expressed as g/L. The Total Soluble Solids measurement is expected to be >90% sugars in grapes [26] and is expressed in units of Brix. Parenthetical values are standard deviations.

**Figure 3:**
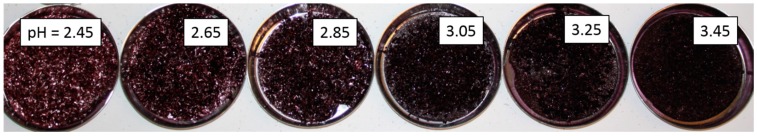
KHT precipitates from Concord juices as a function of pH.

Although the KHT crystals had uniform appearance at high pH, the crystals formed at lower pH were heterogeneous in appearance, with some nearly transparent and others deeply pigmented (Figure 3).

**Figure 4:**
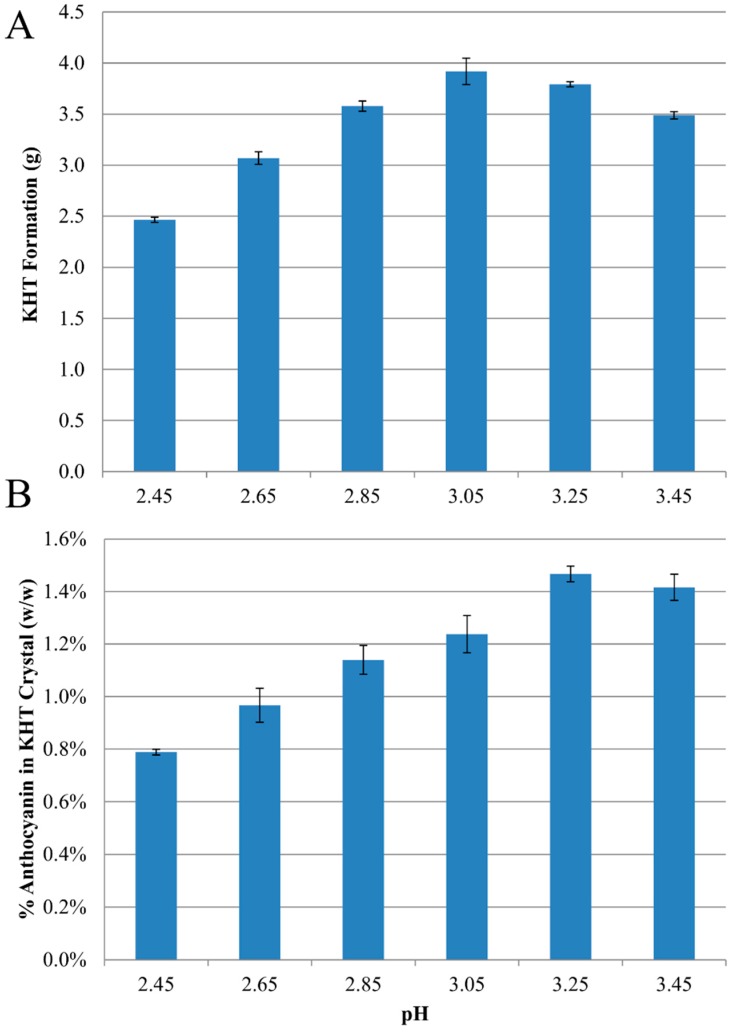
Effects of pH on (**A**) mass (g) of KHT crystals recovered by filtration after cold stabilization of Concord juices, and (**B**) total anthocyanin content (% w/w) of KHT crystals recovered from cold stabilized Concord juice.

As with the model juice, the total mass of KHT precipitate formed decreased with decreasing pH (Figure 4A). For most anthocyanin species, the total mass of each anthocyanin species (in grams) in the final juice and in the precipitate was 90%–110% of the initial mass (Supplementary Figure S1), except for two anthocyanins with 80%–90% recovery (petunidin-3,5-diglucoside and delphinidin-3,5-diglucoside). These two exceptions were challenging to quantify by HPLC due to their low concentrations.

The concentration of anthocyanin in the KHT precipitate was at a maximum at pH 3.25–3.45 (1.4% w/w, Figure 4B), and decreased significantly with decreasing pH below 3.05 to a minimum at pH 2.45 (0.8% w/w). Similar to what was observed with model juice, decreasing the pH to 3.05 or lower resulted in a significant decrease in total anthocyanin coprecipitation, with the lowest content observed at the lowest pH (Figure 4B).

**Figure 5:**
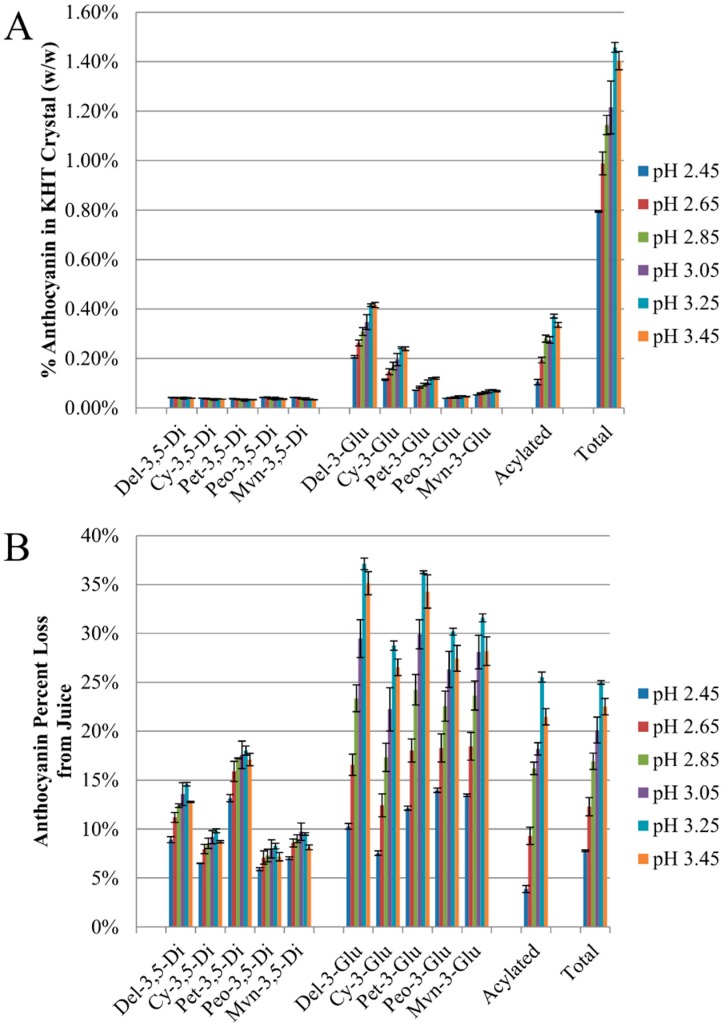

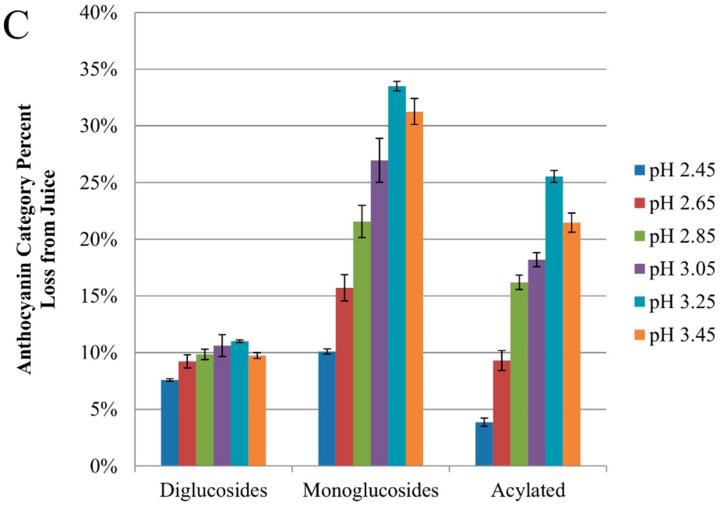
Effects of pH on (**A**) the mass fraction (%w/w) of individual anthocyanins in KHT crystals; (**B**) the percent loss ((Original−Final)/Original ×100%) of individual anthocyanin species lost from Concord juice; and (**C**) the percent loss of anthocyanin classes lost from Concord juice.

The mass fraction of each anthocyanin (%w/w) in KHT crystal (Figure 5A), the percent loss of each anthocyanin from juice (Figure 5B), and the percent loss of each anthocyanin class (Figure 5C) were determined by HPLC.

Monoglucosides accounted for the majority of anthocyanin coprecipitate, with delphinidin-3-glucoside accounting for nearly one-third of the total (Figure 5A). However, when normalized against the original anthocyanin content of each species in the juice, the percent decreases was roughly comparable across all monoglucosides as well as acylated species, with losses in the range of 25%–35% for these species (Figure 5B). Less coprecipitation losses were seen for all monoglucosides at low pH, with losses in the range of 7%–15% at pH 2.45. Small but significant differences in the effect of pH on anthocyanin monoglucoside loss were observed among aglycones. Specifically, the difference in this change was inversely correlated to HPLC retention time on a non-polar column (*p* < 0.05). For example, delphinidin-3-glucoside coprecipitated to a greater extent than malvidin-3-glucoside at high pH, but this trend was reversed at the lowest pH value (Figure 5B). A greater selectivity effect was observed as a result of the glycoside. At pH 3.25 or greater, anthocyanin monoglucosides and acylated forms were enriched in the KHT precipitate as compared to diglucosides (Figure 5C). As the pH decreased below 3, the concentration of anthocyanin monoglucosides and acylated anthocyanins in KHT precipitate also decreased, with maximum differences of 3- to 5-fold at pH 2.45 *vs.* pH 3.25. By comparison, diglucosides showed no significant decrease in anthocyanin content of KHT crystals until pH 2.45, and the difference was less than a factor of 2.

### 2.3. Discussion

Previous work has shown that concentration of juice prior to cold-stabilization decreases the mass fraction of anthocyanins lost to coprecipitation with potassium bitartrate by over four-fold [10]. Because the loss of individual anthocyanin species was correlated with the hydration constant (pK_h_) of the species, it was hypothesized that the decrease in coprecipitation was due to the lower pH of concentrate and resulting neutralization of the bitartrate crystal faces [7]. In exploratory studies, we attempted to use commercial grape anthocyanin extracts in our model juice systems. However, we observed very little coprecipitation of anthocyanins during these preliminary experiments. A possible explanation is that the commercial anthocyanin extract had already undergone cold-stabilization, resulting in the loss of the species most prone to coprecipitation. As an alternative, we chose to use commercially available black currant extract as our source of anthocyanins. Black currants contain four dominant anthocyanins [27] which account for >99% of the total anthocyanin content, which simplifies the chromatographic separation. Two of these anthocyanins are major anthocyanin species in grapes (cyanidin-3-glucoside and delphinidin-3-glucoside), while the other two (cyanidin-3-rutinoside and delphinidin-3-rutinoside) are not observed in grapes.

The low concentrations of the KHT crystal formed at pH < 3 (Figure 1A) were expected due to the low concentration of bitartrate species at pH < 3.0, the pK_a_ of tartaric acid. Interestingly, although the concentration of the bitartrate species is predicted to be at a maximum at pH 3.65 [26], the pH 3.40 model juice did not produce the most KHT precipitate. Instead, the model juices followed the order pH 2.95 > pH 3.2 > pH 3.4 > pH 2.7 > pH 2.35. This is likely due to increasing adsorption of anthocyanins to the growing crystal faces at higher pH. This coprecipitation effect is well known to limit crystal growth and the extent of precipitation [11,16,25].

The maximum anthocyanin concentration observed to coprecipitate (0.19% w/w at pH 3.40) was less than the 0.8% w/w content of KHT recovered from cold stabilization of single strength Concord grape juice [10]. The difference between our model system and Concord may be due to the higher concentrations of both acylated and monoglucoside anthocyanins in Concord, which are more likely to coprecipitate [9,10]. pH had a significant and dramatic effect on anthocyanin loss, with a sharp, order of magnitude decrease in anthocyanin content observed at pH ≤ 2.95 as compared to pH 3.4 (Figure 1B). The observation that a decreased pH results in decreased coprecipitation supports the hypothesis previously advanced by Alongi *et al.* [10] to explain differences in anthocyanin coprecipitation in concentrate and single strength juice. The authors observed that KHT precipitation from juice concentrate resulted in negligible losses as compared to precipitation from single strength juice. The higher pH of juice (3.05) could result in a negative surface charge of KHT and thus increase interactions between the flavylium form of anthocyanins and the deprotonated sites of the bitartrate crystals, while at the low pH of concentrate (2.5) the KHT surface would be neutralized and interactions would diminish. This suggests that the transition from neutral to charged surface occurs around pH 3.0 for KHT. This is below the pH range usually observed for red grape juices and wines, which may explain why this phenomenon had not been previously reported. According to Celotti *et al.* [24], a transition in the surface charge appears to occur between pH 2.8 and 3.0, as measured by streaming potential experiments. Surprisingly, the authors report that the surface charge became more negative with decreasing pH, an observation at odds with what would be expected to occur to surface charge with decreasing pH, and thus may be an error in sign in the earlier publication.

While pH had the major effect on the mass fraction of anthocyanin coprecipitation (over an order of a magnitude), sugar moiety and aglycone also had a smaller but still significant effect. Glucosides were preferentially lost over corresponding rutinosides, and delphinidins were preferentially lost as compared to cyanidins. The reason for the preferential loss of glucosides is unclear, but may be because the rutinosides are sterically hindered as disaccharides and thus less able to adsorb to the KHT crystal surface. The observation that delphinidins preferentially coprecipitated as compared to cyanidins is in contradiction to our previous work [10], where we observed a greater loss of cyanidin-3-glucoside from Concord grape juice (15% decrease) than delphinidin-3-glucoside (3%) from single strength juice during cold stabilization. This unexpected result is discussed in more detail later.

#### 2.3.1. Anthocyanin-Bitartrate Coprecipitation in a Purple Concord Juice

As with the model juice system, decreasing pH resulted in a decrease in total anthocyanin coprecipitation (Figure 5). The decrease in anthocyanin loss did appear to be uniform among KHT crystals, however, and the heterogenous appearance crystals at low pH (Figure 3) may indicate that adsorption of anthocyanins onto the KHT face at pH < 3 involves cooperative binding by anthocyanin species, in which initial adsorption is slow and subsequent binding is faster [28].

The maximum concentration of anthocyanin in the KHT precipitate was at a maximum at pH 3.25 (1.4% w/w, Figure 4B), higher than that observed in previous work on cold stabilization of single strength Concord juice (0.8% w/w, [10]). The higher degree of coprecipitation may be due to the higher concentration of anthocyanins in the current study as opposed to the previous work (492 mg/L *vs.* 278 mg/L as malvidin-3-glucoside equivalents).

Similar to what was observed with model juice, decreasing the pH to < 3.05 resulted in a significant decrease in total anthocyanin coprecipitation, with the lowest content observed at the lowest pH (Figure 4B).

The effect of pH on coprecipitation varied among anthocyanin classes. Anthocyanin monoglucosides and acylated forms were enriched in the KHT precipitate as compared to monoglucosides at higher pH values, pH > 3 (Figure 5A,B). This was in agreement with a previous study which observed the trend acylated > mono- > di- for % w/w anthocyanin in KHT precipitate from unadjusted Concord juice (pH = 3.1) [10], which follows an order of increasing polarity. Similarly, we observed that the loss of monoglucoside anthocyanin species at high pH was inversely correlated to HPLC retention time (*p* < 0.05), suggesting that the likelihood a species would coprecipitate at high pH was correlated to its hydrophobicity and solubility in grape juice. In other words, less soluble species are more likely to coprecipitate with KHT.

An alternate explanation for why monoglucoside and acylated forms of anthocyanins coprecipitate more readily than diglucosides at pH > 3 may be due to changes in KHT surface charge. As with model juice, the sharp decrease in anthocyanin coprecipitation below pH 3 in both model and real juice may relate to the neutralization of KHT surface charge below pH 3.1 [10]. Because diglucosides generally have lower pK_h_ values than their corresponding monoglucosides [29], they will exist in a neutral form to a greater extent and thus be less likely to coprecipitate with the negatively charged KHT surface at high pH. Following KHT surface neutralization at pH < 3, this mechanism is of less importance and under these low pH conditions, coprecipitation would require interaction of neutral anthocyanin species with the neutral KHT crystal face. This would explain why there would be a much larger decrease in monoglucoside and acylated losses (higher pK_h_) with decreasing pH than in diglucosides (lower pK_h_), as shown in Figure 5C. However, in contradiction to this hypothesis, the effect of pH on losses of delphinidin-3-glucoside (pK_h_ = 2.35) and cyanidin-3-glucoside (pK_h_ = 3.01) was comparable (Figure 5B).

The observed decrease in coprecipitation with decreasing pH may partially explain the decrease in coprecipitation observed with juice concentration prior to cold-stabilization. In previous work with 59 Brix concentrate, a 6-fold decrease in co-precipitation was observed (0.8% w/w *vs.* 0.13% w/w) as compared to juice [10]. This is somewhat higher than the ~2-fold decrease observed in the current work. Because diglucoside coprecipitation is not affected by pH, the stronger effect of pH on total anthocyanin loss observed in the earlier work may be partially due to the low concentration of diglucosides (<2% as malvidin-3-glucoside equivalents) as compared to ~25% in the current work. However, other differences exist in juice concentrate (higher ionic strength, higher concentration of K^+^, HT^−^, and anthocyanins) which could also account for the observed effects.

#### 2.3.2. Potential Implications for Juice Processing and Retention or Isolation of Anthocyanins

Our study demonstrates that a lower pH reduces anthocyanin coprecipitation, particularly for acylated and monoglucoside species. The 20%–40% losses of anthocyanins observed during cold stabilization are comparable to gains achieved by more common juice processing treatments like the use of pectolytic enzymes [22], and thus may represent an interesting target for purple grape juice processors to improve color. As a caveat, changes in anthocyanin concentration may not translate into perceivable differences to consumers, since juice color will also depend on other factors like copigmentation and pH [21]. Potentially, coprecipitation losses to anthocyanins during cold-stabilization of grape juice and wine could be eliminated by intentionally reducing the pH below 3 prior to production. Reducing the pH much below 3 is likely undesirable, at least in single strength juice, as insufficient KHT precipitation would occur. A reduction of pH can be achieved by concentration prior to cold-stabilization, as previously demonstrated [10], but this is a complex process, and would not be appropriate for wine or for juices that are intended to be bottled without concentration. Alternatively, the pH could be reduced chemically (*i.e*., by addition of tartaric acid) or by physical means (*i.e*., electrodialysis) prior to cold stabilization. Following cold stabilization, the pH could be raised by analogous chemical or physical processes. Cation-exchange resins could also be used to reduce pH, but these resins are well known to adsorb anthocyanins [30], so an improvement to anthocyanin content probably would not be realized.

Alternatively, coprecipitation with KHT could be exploited to selectively enrich and isolate anthocyanins. Coprecipitation via adsorption is a classic analytical strategy for enriching trace analytes, although the strategy has been used primarily for enriching trace metal cations [31]. In the case of anthocyanins, commercial products are generally sold as crude preparations due to the cost and difficulty of purifying these compounds from complex natural sources [32,33]. As shown here, the concentration of anthocyanins in KHT crystals can exceed 1%, comparable to the loadings achievable with reversed phase resins, but with the advantage that KHT is a fraction of the cost of commercial resins.

## 3. Experimental Section

### 3.1. Chemicals

Black currant powder containing 20% w/w anthocyanin as cyanidin-3-glucoside equivalents was used as an anthocyanin source (Artemis International Inc., Fort Wayne, IN, USA). Malvidin-3-glucose was purchased from Sigma-Aldrich, Inc. (St. Louis, MO, USA). Citric acid monohydrate, malic acid, and anhydrous sodium hydrogen phosphate were purchased from J.T. Baker Chemical Co. (Phillipsburg, NJ, USA). d-Glucose, d-fructose, l-(+)-tartaric acid, potassium chloride, sodium chloride, and 0.01 M hydrochloric acid were purchased from Thermo Fisher Scientific Inc. (Fair Lawn, NJ, USA). Water from a Nanopure water purifier (Barnstead Thermolyne, Boston, MA, USA) was used throughout the study.

### 3.2. Preparation and Cold-Stabilization Treatments of Model Juices

A full factorial design was used to produce model juice systems with varying pH values, K^+^ concentrations, and tartaric acid concentrations. All model juices contained 80 g/kg glucose, 80 g/kg fructose, and 250 mg/L anthocyanin as cyanidin-3-glucoside equivalents (similar to red grape juice). Five pH values were used: 2.35, 2.70, 2.95, 3.20, and 3.40, and were prepared by the appropriate combination of 0.1 M citric acid and 0.2 M sodium hydrogen phosphate buffer solutions. Two K^+^ concentrations were used: 0.02 M and 0.04 M, added in the form of KCl. Two tartaric acid concentrations were used: 0.02 M and 0.04 M. The total number of model juice systems investigated was 5 pH × 2 K^+^ × 2 tartaric = 20 systems. Each juice system was prepared in duplicate. Cold stabilization was performed by storing all model juices at −3 °C for 7 weeks without any bitartrate crystal seeding. The pH of model juices was measured before and after cold stabilization using a pH meter model Orion 3 Star Series pH Benchtop (Thermo Electron Corp., Beverly, MA, USA).

### 3.3. Processing, Preparation, and Cold-Stabilization Treatments of Concord Juice

Frozen deseeded Concord grape mash was obtained from the New York State Agricultural Experiment Station collected from grapes harvested from the Lake Erie region (New York, NY, USA) during the 2013 growing season. The frozen juice was quickly defrosted during the initial stages of kettle pasteurization and diluted to 20.0 Brix, determined by digital refractometry (Misco model #PA203X; Misco Refractometer, Solon, OH, USA). The mash was depectinized with 30 mL of Adex-G (DSM Enzymes, Heerlen, The Netherlands) per 19 L of mash. After bulk pasteurization at 60 °C (140°F) using a 85 L tilting steam-jacketed kettle (model 20CD 1979; Lee Industries, Philipsburg, PA, USA), the juice was immediately pre-filtered through several layers of cheesecloth before being passed through two plate filter beds packed with 0.75% (w/w) Celite 503 (Imerys Filtration Minerals, San Jose, CA, USA) using a size 7 Shriver plate and frame filter press (FLSmidth, Salt Lake City, UT, USA).

Six equal aliquots of juice were pH adjusted from the base pH of 3.05 using either 1 M HCl or 1 M NaOH as required to form the pH 2.45, 2.65, 2.85, 3.05, 3.25, and 3.45 sample series. Following adjustment, and prior to cold stabilization, samples were analyzed for soluble solids by refractometry, organic acids by HPLC [34], and anthocyanins. Triplicate 500 mL portions of each pH-adjusted juice were transferred to autoclave-sterilized 500 mL Pyrex storage bottles (Thermo Fisher Scientific, Waltham, MA, USA) and cold stabilized for three weeks at −3 °C, shielded from light.

### 3.4. Characterization of Anthocyanins in KHT Crystals

KHT crystals were collected by filtration on a glass fiber filter (Type A/E, PALL Corp, Ann Arbor, MI, USA), followed by a washing step with cold 95% ethanol to remove any loosely adhering material on the crystal surface. The crystals were dried to constant weight in an oven at 60 °C and weighed prior to anthocyanin analysis.

#### 3.4.1. KHT Solubilization

For the blackcurrant model juice samples, 50 mg of KHT crystals were dissolved at room temperature in 3 mL of 1M NaCl acidified with HCl (0.01 M). When less than 50 mg of precipitate was formed, a proportionally reduced volume of the acidified NaCl solution was used for dissolution. After dissolution (approximately 15 min) each sample was immediately filtered through a 0.2 µm regenerated cellulose membrane (Sigma-Aldrich, St. Louis, MO, USA) in preparation for HPLC analysis. The KHT crystals recovered from the Concord juice sample set were prepared in a similar fashion with the exception that the entire recovered portion of KHT crystal was dissolved in 500 mL of the NaCl/HCl solution.

#### 3.4.2. HPLC Analysis of Anthocyanins in Model Juice Samples

For the blackcurrant samples, resuspended anthocyanins were analyzed using an Agilent 1100 series HPLC system with inline degasser, autosampler and diode array detector (Agilent Technologies, Santa Clara, CA, USA). A 250 mm × 4.6 mm Varian LiChrospher RP-18 endcapped column (particle size 5 µm, pore size 100 Å; Varian, Inc., Palo Alto, CA, USA) was maintained at 30 °C by an Eppendorf CH-30 external column heater. A 50 µL aliquot of each sample was injected on to the HPLC system. Mobile phase A consisted of water/phosphoric acid (99.5:0.5) and mobile phase B consisted of acetonitrile/water/phosphoric acid (50:49.5:0.5). Analytes of interest were resolved over a 38 min gradient elution profile starting at 0% B for 2 min, increasing to 20% B over 5 min, increasing to 36% B over 15 min, increasing to 100% B over 6 min, holding at 100% B for 2 min, followed by an 8 min return to starting conditions. The flow rate was 1 mL/min. The eluent was monitored at 520 nm. Analytes were identified based on comparison of relative retention times to those previously reported for anthocyanins in blackcurrant juice [27]. Quantification of each anthocyanin was based on a malvidin-3-glucose standard curve and thus reported in units of malvidin-3-glucoside equivalents, as is common for anthocyanin analyses [35]. The total anthocyanin content was calculated as the sum of all major anthocyanins identified by HPLC analysis and expressed in units of malvidin-3-glucoside equivalents. Two analytical replicates were performed on each sample.

#### 3.4.3. HPLC Analysis of Anthocyanins in Real Juice Samples

Due to the presence of diglucosidic forms, anthocyanins from the Concord juice sample set were resolved using a 100 mm × 2.1 mm ID (2.6 µm particle size) Kinetex pentafluorophenyl (PFP) column (Phenomenex, Torrance, CA, USA) attached to an Agilent 1260 HPLC system equipped with an inline degasser, autosampler, thermostated column compartment, and diode array detector and analyzed using a previously published method [36]. Retention times had been previously demonstrated to not vary significantly among juice samples and standards. Monoglucosides and modified anthocyanins (acylated, methylated, *etc.*) were quantified using a malvidin-3-glucoside standard while diglucosides were quantified using a malvidin-3,5-diglucoside standard. The stability of the anthocyanins in the acidified NaCl solution preparation was evaluated by repeatedly analyzing one sample from each pH solution at 0, 24, 48, 72 h. No significant differences were observed.

### 3.5. Statistical Analysis

Results were reported in mean ± standard deviation. Data were subjected to analysis of variance (ANOVA). For treatments with a significant effect, means were compared with Tukey-Kramer HSD at 95% confidence interval using the JMP^®^ 8.0 statistical software package (SAS institute Inc., Cary, NC, USA).

## 4. Conclusions

At pH ≤ 3, potassium bitartrate removal by cold stabilization of both real and model juices results in significantly less coprecipitation of anthocyanins with KHT crystals. This is likely due to changes in the charged of the KHT crystal surface, and may explain a previous observation that cold-stabilization of concentrate results in negligible coprecipitation. Potentially, pH adjustments could be made to juice by chemical or physical processes prior to cold stabilization to reduce losses of anthocyanins. Acylated and monoglucosides coprecipitate to a much greater extent than diglucosides at pH > 3, but this effect was minimized at low pH. Other minor selectivity effects were observed due to the aglycone.

## Supplementary Materials

Supplemental Figure S1: Mass balance of anthocyanin species before and after cold stabilization. Supplementary materials can be accessed at: http://www.mdpi.com/1420-3049/20/01/0556/s1.
