# Supplementary material for: Decreasing pH Results in a Reduction of Anthocyanin Coprecipitation during Cold Stabilization of Purple Grape Juice
Source: Molecules. 2015 Jan 5;20(1):556–72. doi: 10.3390/molecules20010556 (PMC6272568; doi:10.3390/molecules20010556)
Supplement: Supplementary file 1 [file molecules-20-00556-s001.pdf]

Supplementary Materials

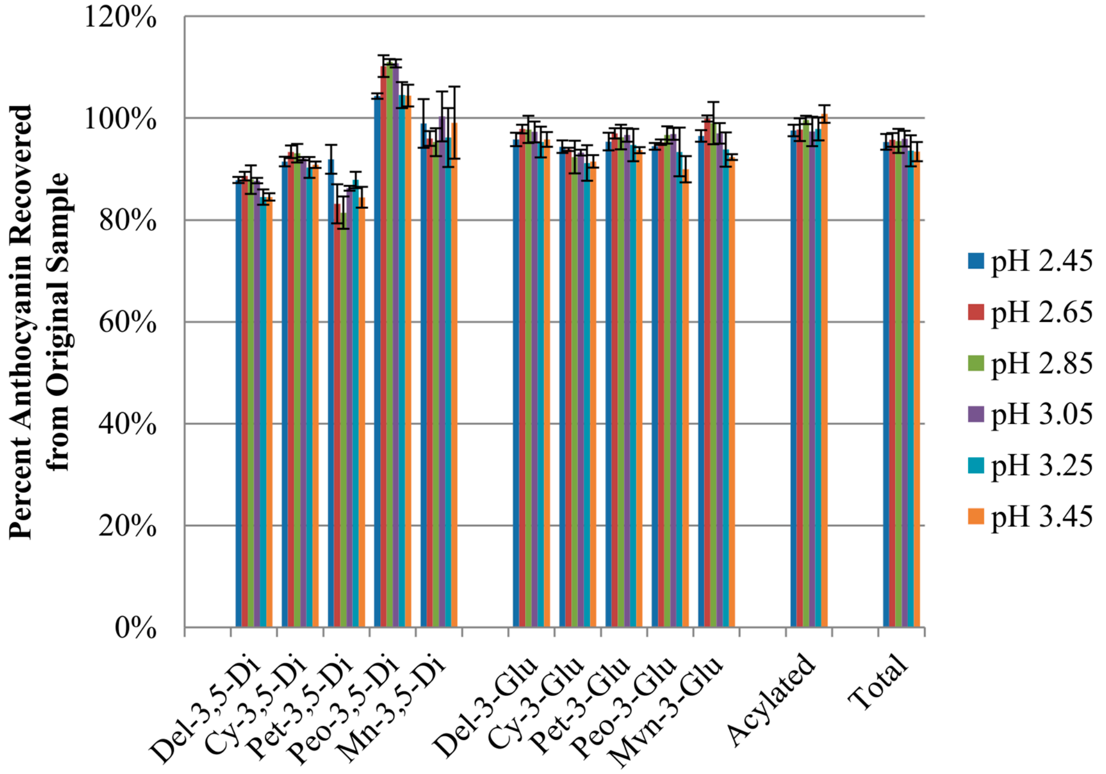

Figure S1. Mass balance of anthocyanin species. The recovery was calculated as the ratio of the sum of anthocyanins in KHT crystals and in the cold-stabilized juice to the initial mass of anthocyanin in the juice prior to cold stabilization.
